# Hypertension is prevalent in non-alcoholic fatty liver disease and increases all-cause and cardiovascular mortality

**DOI:** 10.3389/fcvm.2022.942753

**Published:** 2022-08-08

**Authors:** Cheng Han Ng, Zhen Yu Wong, Nicholas W. S. Chew, Kai En Chan, Jieling Xiao, Nilofer Sayed, Wen Hui Lim, Darren Jun Hao Tan, Ryan Wai Keong Loke, Phoebe Wen Lin Tay, Jie Ning Yong, Gywneth Kong, Daniel Q. Huang, Jiong-Wei Wang, Mark Chan, Mayank Dalakoti, Nobuharu Tamaki, Mazen Noureddin, Mohammad Shadab Siddiqui, Arun J. Sanyal, Mark Muthiah

**Affiliations:** ^1^Yong Loo Lin School of Medicine, National University of Singapore, Singapore, Singapore; ^2^School of Medicine, International Medical University, Kuala Lumpur, Malaysia; ^3^Department of Cardiology, National University Heart Centre, National University Hospital, Singapore, Singapore; ^4^Department of Surgery, Yong Loo Lin School of Medicine, National University of Singapore, Singapore, Singapore; ^5^Nanomedicine Translational Research Programme, Centre for NanoMedicine, Yong Loo Lin School of Medicine, National University of Singapore, Singapore, Singapore; ^6^Division of Gastroenterology and Hepatology, Department of Medicine, National University Hospital, Singapore, Singapore; ^7^National University Centre for Organ Transplantation, National University Health System, Singapore, Singapore; ^8^Cardiovascular Research Institute (CVRI), National University Heart Centre Singapore (NUHCS), Singapore, Singapore; ^9^Department of Physiology, Yong Loo Lin School of Medicine, National University of Singapore, Singapore, Singapore; ^10^Department of Gastroenterology and Hepatology, Musashino Red Cross Hospital, Tokyo, Japan; ^11^Cedars-Sinai Fatty Liver Program, Division of Digestive and Liver Diseases, Department of Medicine, Comprehensive Transplant Center, Cedars-Sinai Medical Centre, Los Angeles, CA, United States; ^12^Division of Gastroenterology, Hepatology and Nutrition, Department of Internal Medicine, Virginia Commonwealth University, Richmond, VA, United States

**Keywords:** non-alcoholic fatty liver (NAFL), controlled hypertension, uncontrolled hypertension, mortality, cardiovascular

## Abstract

**Background and aims:**

Hypertension (HTN) is a common comorbidity in non-alcoholic fatty liver disease (NAFLD) affecting up to 40% of individuals. However, the impact of HTN and its control on outcomes in NAFLD remains unclear. Therefore, we aimed to examine the impact of HTN on survival outcomes in a longitudinal cohort of NAFLD patients.

**Methods:**

The analysis consisted of adults in the National Health and Nutrition Examination Survey (NHANES) from 1999 to 2018 with data on socio-demographic characteristics and comorbidities. NAFLD was diagnosed with fatty liver index (FLI) and United States-FLI at a cut-off of 60 and 30, respectively in the substantial absence of alcohol use. A multivariate regression analysis was conducted to adjust for confounders.

**Results:**

A total of 45,302 adults were included, and 27.83% were identified to have NAFLD. Overall, 45.65 and 35.12% of patients with NAFLD had HTN and uncontrolled HTN, respectively. A multivariate analysis with confounders demonstrated that hypertensive NAFLD had a significantly increased risk of all-cause mortality (HR: 1.39, CI: 1.14–1.68, *p* < 0.01) and cardiovascular disease (CVD) mortality (HR: 1.85, CI: 1.06–3.21, *p* = 0.03). Untreated HTN remained to have a significantly increased risk in all-cause (HR: 1.59, CI: 1.28–1.96, *p* < 0.01) and CVD mortality (HR: 2.36, CI: 1.36–4.10, *p* < 0.01) while treated HTN had a non-significant increased risk of CVD mortality (HR: 1.51, CI: 0.87–2.63, *p* = 0.14) and a lower magnitude of increase in the risk of all-cause mortality (HR: 1.26, CI: 1.03–1.55, *p* = 0.03).

**Conclusion:**

Despite the significant burden of HTN in NAFLD, up to a fifth of patients have adequate control, and the lack thereof significantly increases the mortality risk. With the significant association of HTN in NAFLD, patients with NAFLD should be managed with a multidisciplinary team to improve longitudinal outcomes.

## Introduction

Non-alcoholic fatty liver disease (NAFLD) affects ≈25–33% of the global population and represents a spectrum consisting of non-alcoholic fatty liver (NAFL) and non-alcoholic steatohepatitis (NASH) which impose significant healthcare and economic burden globally (1–3). NAFLD is the leading cause of cirrhosis, liver disorders, and liver-related mortality (4–6). The increased fat deposition in hepatocytes results from a myriad of factors including insulin resistance, lipotoxicity, and changes in gut microbiota can increase hepatic and extrahepatic complications (7–9). Several epidemiological studies have shown that NAFLD can lead to an increment in all-cause mortality and is significantly associated with the development of hypertension (HTN), cardiovascular diseases (CVDs), hepatocellular carcinoma, diabetes mellitus, and renal and lung diseases (6, 10–15).

Hypertension (HTN) accounted for 8.5 million deaths in 2015 alone (16, 17) and is a known risk factor associated with NAFLD (13, 18). In a meta-analysis by Ciardullo et al. of 11 longitudinal studies, patients with NAFLD were found to have an increased risk of HTN by 66% (HR: 1.66, CI: 1.38–2.01) (19). While the coexistence of HTN and NAFLD is widely recognized, however, the exact cause-effect relationship and pathophysiological mechanism of NAFLD and HTN remain elusive (18, 20). Current evidence has however demonstrated that the pretext of HTN was associated with promoting the development of NAFLD to its most severe forms, including NASH, and most importantly, advanced liver fibrosis, cirrhosis, and hepatocellular carcinoma (21–23). However, the longitudinal risk of all-cause mortality and CVD mortality remains to be examined in NAFLD. In view of the significant global burden of NAFLD and its intrinsic link with HTN, it is necessary to clarify the role of HTN control in affecting the prognosis of NAFLD. This study, therefore, aims to compare the survival outcomes for cohorts of patients with NAFLD who have controlled HTN and uncontrolled HTN with those who are HTN-free, extracting data from the National Health and Nutrition Examination Survey (NHANES) database with longitudinal data from the National Death Index (NDI).

## Methods

### Study population

The aggregated data from NHANES between 1999 and 2018 were analyzed. Briefly, the NHANES study is a stratified and clustered sampled national survey of health and health-related information that includes individuals who are representative of the general, non-institutionalized United States population. It consists of a comprehensive interview, medical examination, and laboratory assessments. A mortality analysis was conducted by linking the data from NHANES to death certificates from the NDI. The information used for this study was conducted and published publicly by the National Center for Health Statistics (NCHS). The present analysis was exempted from Institutional Review Board due to the anonymous nature of the data.

### Definitions

The current study defined HTN as the presence of blood pressure ≥140/90 mmHg or the use of any antihypertensive medications at a population level in accordance with the 2020 International Society of Hypertension (ISH) Global Hypertension Practice Guidelines (24) and the 2019 guidelines of the American College of Cardiology (ACC)/American Heart Association (AHA) on the Primary Prevention of Cardiovascular Diseases (25). Hypertensive medications include angiotensin-converting enzyme inhibitors (ACEI), angiotensin receptor blockers (ARB), beta-blockers (BB), diuretics, alpha-blockers, calcium channel blockers, and other less commonly used antihypertensives. A sensitivity analysis was conducted to stratify patients into controlled and uncontrolled HTN. Patients with controlled HTN were defined as with the presence of antihypertensive use and blood pressure of <140/90 mmHg, and uncontrolled HTN was defined as high blood pressure even with antihypertensive use, in line with the latest practice guidelines available (24, 25). The average blood pressure values were taken from a mean of two systolic and diastolic readings. In accordance with the American Association for the Study of Liver Disease (AASLD) guidelines for NAFLD (26), the diagnosis of NAFLD was made if the following criteria were fulfilled including (1) Evidence of hepatic steatosis using fatty liver index (FLI)/United States Fatty Liver Index (US-FLI), (2) Lack of substantial alcohol consumption ( ≤ 2 drinks/day in men, ≤ 3 drinks/day in women). An FLI ≥ 60 (27) or US-FLI ≥ 30 (28) indicates the presence of hepatic steatosis which has a corresponding area under the receiver operated characteristic (AUROC) of 0.834 (29). Quantification of fibrosis in the liver was examined using Fibrosis-4 (FIB-4), which has an AUROC of 0.871 (30). Diabetes was defined if any of the following conditions were met: (1) A physician diagnosis of diabetes, (2) fasting plasma glucose ≥7mmol/l and glycohemoglobin ≥6.5%, and (3) treatment with any oral hypoglycaemic agents or insulin. HTN was identified for any individual with systolic blood pressure ≥140, diastolic blood pressure ≥90, or the use of antihypertensive therapy (31). The estimated glomerular filtration rate (eGFR) was computed using the Modification of Diet in Renal Disease (MDRD) equation, and any individual with either kidney damage or eGFR ≤ 60 ml/min/1.73 m^2^ was defined as having chronic kidney disease (CKD) (32).

### Statistical analysis

All statistical analysis was conducted in STATA (16.1). Continuous variables were examined either with the Wilcoxon ranked sum test or the Kruskal–Wallis analysis in the context of two or more groups while binary variables were examined with the chi-square test. To examine the magnitude of effect, a generalized linear model with a log link and robust variance estimator clustered on the year of study was used to examine the binary events including the risk and risk factors of HTN in NAFLD (33). When events are common, the risk ratio is a more appropriate approximation of events and provides better interpretability compared to odds ratios (34). Survival analysis was conducted with the Cox proportional model for all-cause mortality for hazard ratios (HR) and analysis of CVD mortality was conducted with the Fine-Gray subdistribution hazard ratio (SHR) to account for competing risk. Univariate and multivariate models were conducted with clustering on the year of study to account for heterogeneity between different year groups. Two multivariate models were conducted in all analyses of risk ratio (RR), HR, and SHR. In model 1, covariates including age, gender, body mass index (BMI), ethnicity, and diabetic status were included in the analysis. Model 2 included smoking status and FIB-4 in addition to variables in model 1.

## Results

In total, there were 41,685 patients included in the analysis, of which 11,435 had NAFLD (27.43%). A total of 9,727 individuals with NAFLD had measurements of blood pressure. Patients with NAFLD were generally older and more likely to be diabetic compared to non-NAFLD individuals (Table 1). The summary of baseline characteristics between NAFLD and non-NAFLD individuals is summarized in Table 1. The prevalence of HTN among individuals with NAFLD was 45.65%. The unadjusted effect size from a generalized linear model with a robust variance estimator clustered on the study period shows that NAFLD was associated with a 48% increased risk of HTN in NAFLD (RR: 1.48, CI: 1.38–1.58, *p* < 0.01). After adjusting for confounders, there remained a statistically significant increase in the risk of HTN and NAFLD in both models 1 and 2, respectively (RR: 1.06, CI: 1.03–1.09, *p* < 0.01 and RR: 1,05, CI: 1.02–1.08, *p* < 0.01). NALFD individuals were more likely to have uncontrolled HTN compared to those without NAFLD (*p* < 0.01). Unsurprisingly, individuals with NAFLD were also found to have significantly poorer lipid profiles (*p* < 0.01), be diabetic (*p* < 0.01), have higher median BMI (*p* < 0.01), and have higher weight circumference (*p* < 0.01). In Table 2, NAFLD individuals with HTN were generally older, diabetic, and more likely to have a higher median BMI. NAFLD individuals with HTN have a higher tendency to be active smokers compared to those without HTN.

**Table 1:** Baseline characteristics between NAFLD vs. non-NAFLD.

	**NAFLD**	**Non-NAFLD**	***P* value**
Age	52.57 (IQR: 39.00–66.00)	43.98 (IQR: 27.00–60.00)	<0.01^*^
Gender			
Male	0.44 (CI: 0.43–0.45)	0.49 (CI: 0.48–0.49)	<0.01^*^
Female	0.56 (CI: 0.55–0.57)	0.51 (CI: 0.51–0.52)	
Ethnicity			
Mexican American	0.20 (CI: 0.19–0.21)	0.19 (CI: 0.18–0.19)	<0.01^*^
Other Hispanic	0.08 (CI: 0.07–0.08)	0.08 (CI: 0.08–0.09)	
Caucasian	0.44 (CI: 0.43–0.45)	0.43 (CI: 0.43–0.44)	
African American	0.21 (CI: 0.21–0.22)	0.19 (CI: 0.19–0.20)	
Others	0.07 (CI: 0.06–0.07)	0.10 (CI: 0.10–0.11)	
Body mass index	34.03 (IQR: 29.73–36.97)	26.64 (IQR: 22.90–29.20)	<0.01^*^
Waist circumference	111.21 (IQR: 102.50–117.90)	92.73 (IQR: 82.40–100.50)	<0.01^*^
Weight	94.59 (IQR: 81.00–104.80)	74.95 (IQR: 62.30–84.00)	<0.01^*^
Diabetes	0.26 (CI: 0.25–0.27)	0.10 (CI: 0.10–0.11)	<0.01^*^
HTN	0.46 (CI: 0.45–0.47)	0.23 (CI: 0.23–0.24)	<0.01^*^
HTN Status			
No HTN	0.54 (CI: 0.53–0.55)	0.77 (CI: 0.76–0.77)	<0.01^*^
HTN controlled with medication	0.30 (CI: 0.29–0.31)	0.15 (CI: 0.14–0.15)	<0.01^*^
HTN uncontrolled with medication	0.16 (CI: 0.15–0.17)	0.08 (CI: 0.08–0.09)	<0.01^*^
Platelet count	258.86 (IQR: 211.00–298.00)	252.63 (IQR: 209.00–289.00)	<0.01^*^
Glycohemoglobin	5.99 (IQR: 5.30–6.10)	5.53 (IQR: 5.10–5.70)	<0.01^*^
Fasting glucose	6.55 (IQR: 5.38–6.68)	5.71 (IQR: 5.00–5.83)	<0.01^*^
Total bilirubin	10.52 (IQR: 6.84–13.68)	11.66 (IQR: 8.55–13.68)	<0.01^*^
Total cholesterol	199.48 (IQR: 170.00–225.00)	190.48 (IQR: 161.00–215.00)	<0.01^*^
LDL	117.25 (IQR: 92.00–139.00)	112.20 (IQR: 87.00–133.00)	<0.01^*^
HDL	47.68 (IQR: 39.00–55.00)	55.30 (IQR: 44.00–64.00)	<0.01^*^
Triglycerides	191.97 (IQR: 111.00–232.00)	128.45 (IQR: 69.00–152.00)	<0.01^*^
eGFR	91.67 (IQR: 74.83–110.69)	99.75 (IQR: 82.97 to119.07)	<0.01^*^
Smoking status			
Non smokers	0.60 (IQR: 0.59–0.61)	0.55 (IQR: 0.54–0.56)	<0.01^*^
Past smokers	0.27 (IQR: 0.26–0.28)	0.23 (IQR: 0.22–0.24)	
Current smokers	0.13 (0.12–0.13)	0.22 (IQR: 0.21–0.22)	

* Bolded p-value ≤ 0.05 denotes statistical significance. LDL, Low-Density Lipoprotein; HDL, High-Density Lipoprotein; HTN, Hypertension; eGFR, Estimated Glomerular Filtration Rate; IQR, Interquartile Range; CI, Confidence Interval.

**Table 2:** Comparisons between non hypertensive, controlled and uncontrolled HTN in NAFLD.

	**No HTN**	**Controlled HTN**	**Uncontrolled HTN**	***P* value**
Age	43.55 (IQR: 31.00–54.00)	61.82 (IQR: 54.00–71.00)	65.23 (IQR: 58.00–75.00)	<0.01^*^
Gender				
Male	0.46 (CI: 0.44–0.47)	0.46 (CI: 0.44–0.47)	0.42 (CI: 0.39–0.44)	0.03^*^
Female	0.54 (CI: 0.53–0.56)	0.54 (CI: 0.53–0.56)	0.58 (CI: 0.56–0.61)	
Ethnicity				
Mexican American	0.25 (CI: 0.24–0.26)	0.13 (CI: 0.12–0.14)	0.15 (CI: 0.14–0.17)	<0.01^*^
Other hispanic	0.09 (CI: 0.08–0.10)	0.07 (CI: 0.06–0.08)	0.07 (CI: 0.06–0.09)	
Caucasian	0.41 (CI: 0.39–0.42)	0.51 (CI: 0.49–0.53)	0.45 (CI: 0.42–0.47)	
Black	0.17 (CI: 0.16–0.18)	0.23 (CI: 0.21–0.25)	0.27 (CI: 0.25–0.29)	
Other race	0.08 (CI: 0.07–0.09)	0.06 (CI: 0.05–0.07)	0.06 (CI: 0.05–0.07)	
Body mass index	33.72 (IQR: 29.66–36.52)	34.30 (IQR: 29.90–37.40)	33.72 (IQR: 29.36–36.90)	<0.01^*^
Waist circumference	109.51 (IQR: 101.30–115.80)	113.40 (IQR: 104.50–120.30)	111.79 (IQR: 102.90–118.70)	<0.01^*^
Weight	94.35 (IQR: 81.50–104.40)	95.53 (IQR: 81.90–105.30)	92.28 (IQR: 77.60–103.90)	<0.01^*^
Diabetes	0.13 (CI: 0.12–0.14)	0.42 (CI: 0.40–0.44)	0.42 (CI: 0.40–0.45)	<0.01^*^
Platelet count	263.14 (IQR: 216.00–301.00)	249.34 (IQR: 200.00–287.00)	248.85 (IQR: 203.00–284.50)	<0.01^*^
Glycohemoglobin	5.73 (IQR: 5.20–5.80)	6.28 (IQR: 5.50–6.60)	6.32 (IQR: 5.60–6.60)	<0.01^*^
Fasting glucose	6.12 (IQR: 5.22–6.11)	6.92 (IQR: 5.61–7.33)	7.22 (IQR: 5.66–7.65)	<0.01^*^
Total bilirubin	10.16 (IQR: 6.84–11.97)	11.03 (IQR: 6.84–13.68)	10.69 (IQR: 6.84–13.68)	<0.01^*^
Total cholesterol	203.43 (IQR: 175.00–227.00)	190.81 (IQR: 161.00–217.00)	199.71 (IQR: 169.00–227.00)	<0.01^*^
LDL	122.18 (IQR: 98.00–142.00)	109.79 (IQR: 84.00–131.00)	113.89 (IQR: 87.00–138.00)	<0.01^*^
HDL	46.84 (IQR: 38.00–54.00)	48.10 (IQR: 39.00–55.00)	48.80 (IQR: 40.00–56.00)	<0.01^*^
Triglycerides	195.65 (IQR: 110.00–237.00)	188.37 (IQR: 112.00–224.00)	189.41 (IQR: 115.00–235.00)	0.17
eGFR	81.20 (IQR: 64.25–98.41)	80.01 (IQR: 65.42–96.93)	101.21 (IQR: 84.94–120.48)	<0.01^*^
Smoking status				
Non smokers	0.58 (IQR: 0.55–0.60)	0.55 (IQR: 0.53–0.57)	0.63 (IQR: 0.62–0.64)	<0.01^*^
Past smokers	0.34 (IQR: 0.31–0.36)	0.34 (IQR: 0.33–0.36)	0.21 (IQR: 0.20–0.23)	
Current smokers	0.08 (IQR: 0.07–0.10)	0.10 (IQR: 0.096–0.12)	0.15 (IQR: 0.14–0.16)	

* Bolded p-value ≤ 0.05 denotes statistical significance.

HTN, Hypertension; LDL, Low-Density Lipoprotein; HDL, High-Density Lipoprotein; eGFR, Estimated Glomerular Filtration Rate; IQR, Interquartile Range; CI, Confidence Interval.

**Figure 1:**
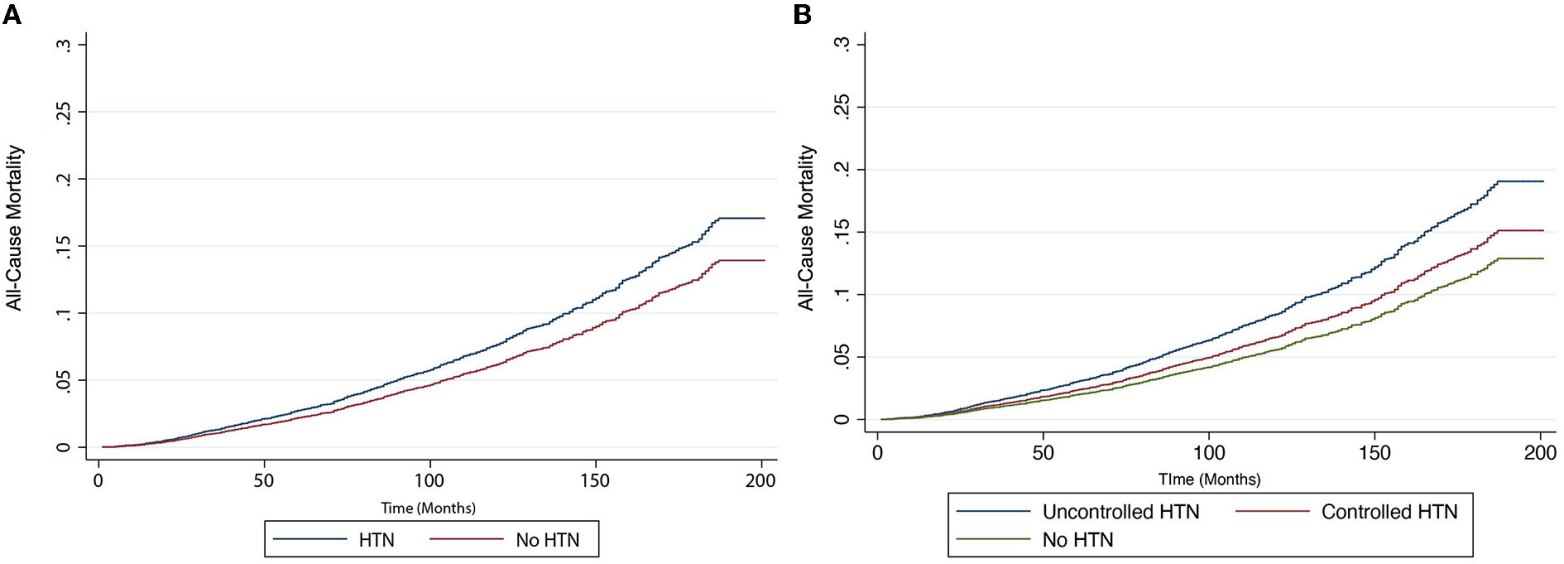
Risk of all-cause mortality in individuals with non-alcoholic fatty liver disease (NAFLD). **(A)** with and without hypertension and **(B)** with controlled hypertension, uncontrolled hypertension, and no hypertension.

**Figure 2:**
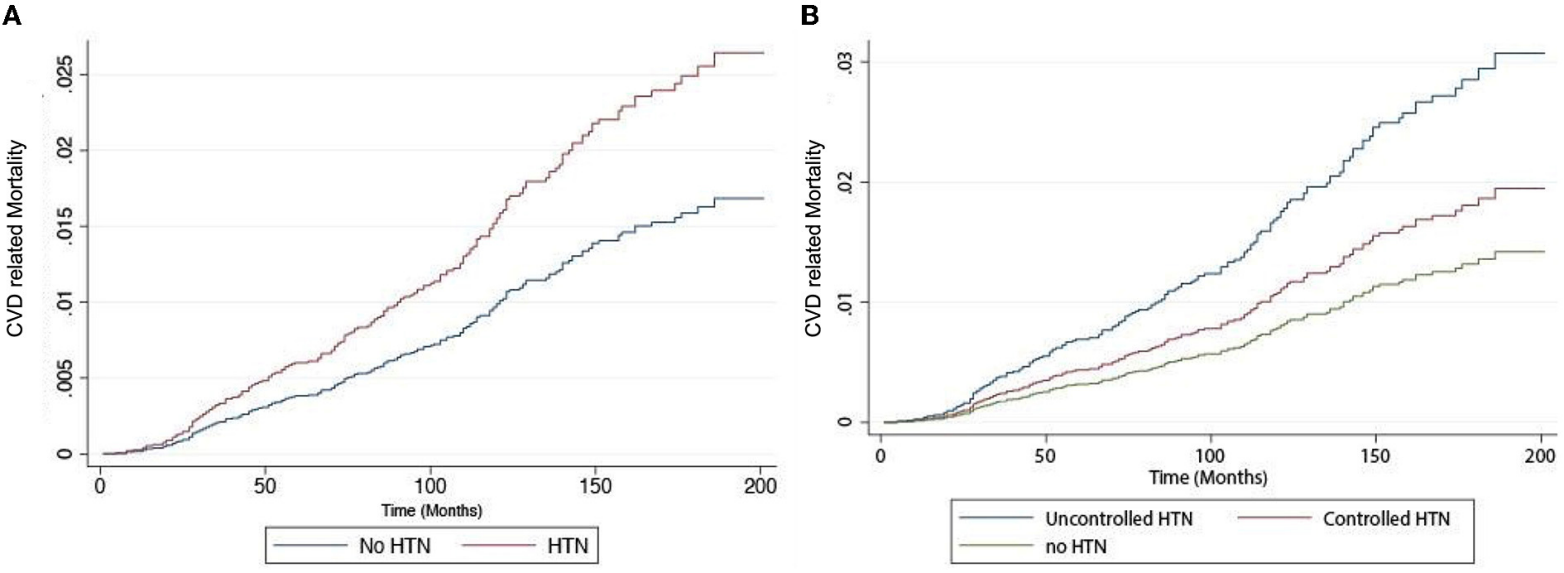
Risk of cardiovascular mortality in individuals with non-alcoholic fatty liver disease (NAFLD). **(A)** with and without hypertension and **(B)** with controlled hypertension, uncontrolled hypertension, and no hypertension.

In an unadjusted analysis, there was a statistically significant relationship in all-cause and CVD-related mortality between HTN and NAFLD (Table 3). We conducted two multivariate models to adjust for confounders. In model 1, covariates including age, gender, BMI, ethnicity, and diabetic status were included in the analysis. There was a statistically significant increase in all-cause mortality (HR: 1.35, CI: 1.12–1.63, *p* < 0.01) and cardiovascular-related mortality (HR: 1.77, CI: 1.03–3.05, *p* = 0.04) in NAFLD individuals with HTN. A second model was conducted to include smoking status and FIB-4 in addition to variables in model 1. There was similarly an increase in all-cause mortality (HR: 1.39, CI: 1.14–1.68, *p* < 0.01, Figure 1) and CVD-related mortality (HR: 1.85, CI: 1.06–3.21, *p* = 0.03, Figure 2A) in NAFLD individuals with HTN.

**Table 3:** All-cause mortality and cardiovascular related mortality in individuals with hypertension, treated hypertension and untreated hypertension.

	**All-cause**			**Cardiovascular**		
	**mortality**			**related mortality**		
	**Unadjusted**	**Model 1**	**Model 2**	**Unadjusted**	**Model 1**	**Model 2**
All HTN	4.71 (CI: 3.98–5.58)*, ***p*** **<** **0.01**	1.35 (CI: 1.12–1.63)*, ***p*** **<** **0.01**	1.39 (CI: 1.14–1.68)*, ***p*** **<** **0.01**	6.13 (CI: 4.17–9.01)*, ***p*** **<** **0.01**	1.77 (CI: 1.03–3.05)*, ***p*** **=** **0.04**	1.85 (CI: 1.06–3.21)*, ***p*** **=** **0.03**
Treated HTN	3.91 (CI: 3.25–4.70)*, ***p*** **<** **0.01**	**1.22 (CI: 1.00–1.50)**, ***p*** **<** 0.01*	1.26 (CI: 1.03–1.55)*, ***p*** **=** **0.03**	4.62 (CI: 3.00–7.10)*, ***p*** **<** **0.01**	1.46 (CI: 0.84–2.55), ***p*** **=** 0.18	1.51 (CI: 0.87–2.63), *P* = 0.14
Untreated HTN	6.04 (CI: 5.01–7.28)*, ***p*** **<** **0.01**	1.56 (CI: 1.26–1.92)*, ***p*** **<** **0.01**	1.59 (CI: 1.28–1.96)*, ***p*** **<** **0.01**	8.57 (CI: 6.12–12.00)*, ***p*** **<** **0.01**	2.23 (CI: 1.30–3.81)*, ***P*** **<** **0.01**	2.36 (CI: 1.36–4.10)*, ***P*** **<** **0.01**

The asterisks and bold values indicate statistical significance values.

HTN, Hypertension; CI, Confidence Interval; BMI, Body Mass Index; FIBto4, Fibrosisto4 Index.

Model 1—Adjusted for age, gender, BMI, ethnicity, diabetic status.

Model 2—Adjusted for Model 1 + smoking status, FIBto4 scores.

**Figure 3:**
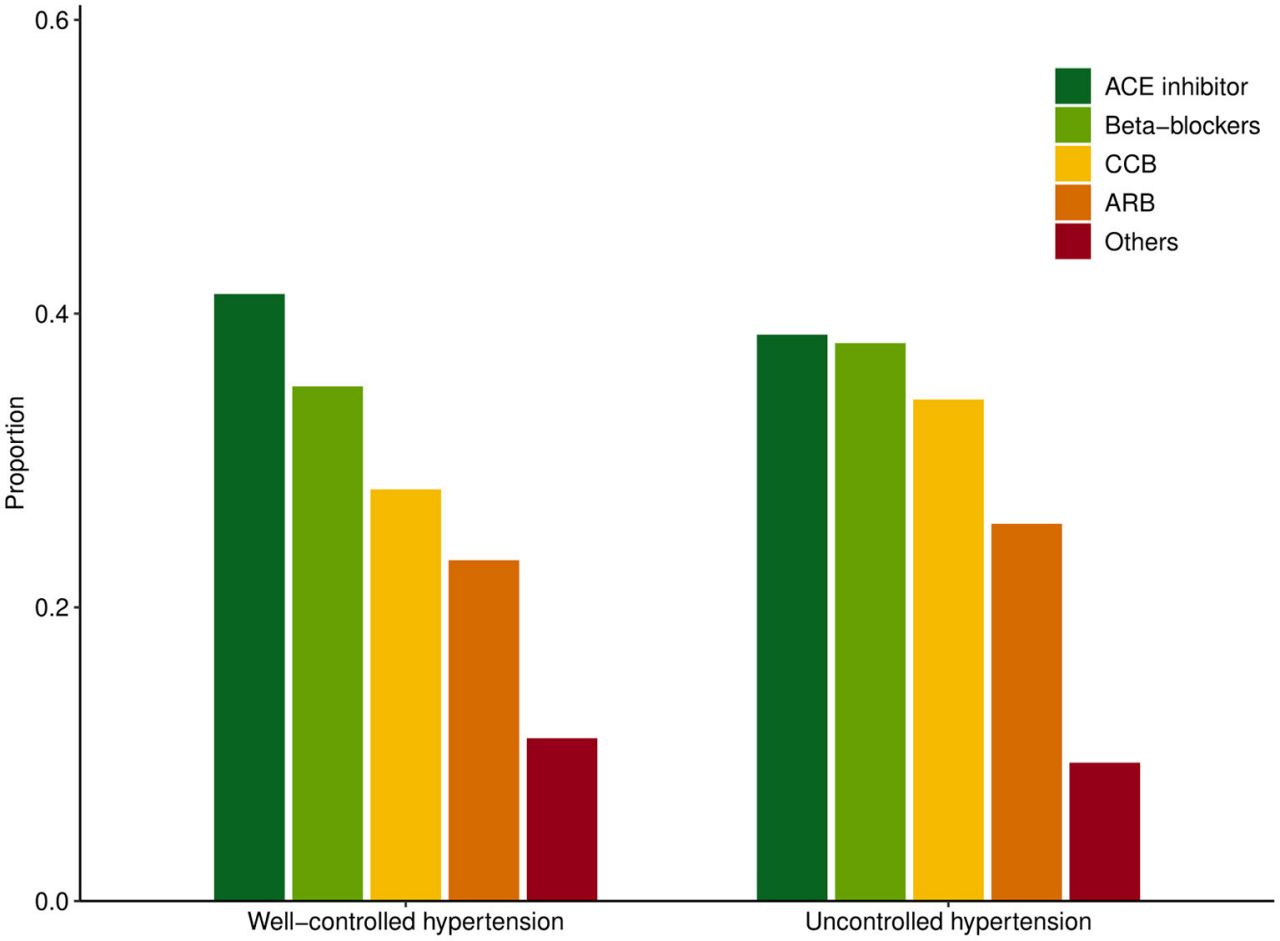
Distribution of anti-hypertensives used in well-controlled hypertension and uncontrolled hypertension.

A sensitivity analysis was then conducted based on hypertensive control and patients were stratified into (1) no HTN, (2) controlled HTN, (3) and uncontrolled HTN. The summary of baseline characteristics can be found in Table 2. Among the NAFLD patients with HTN, 65.84% of patients were adequately controlled while 35.12% were considered uncontrolled HTN. A total of 40.73% of patients were prescribed ACEI, 24.07% had ARB, 36.41% had BB, and 30.45% had calcium channel blockers. The breakdown of medications in patients with well-controlled HTN and uncontrolled HTN can be found in Figure 3. There was a statistically significant relationship between all-cause and CVD-related mortality in both treated and untreated HTN in unadjusted analysis (Table 3). After adjusting for variables in model 1, there remained a statistically significant increase in all-cause (HR: 1.56, CI: 1.26–1.92, *p* < 0.01) and CVD-related mortality (HR: 2.23, CI: 1.30–3.81, *p* < 0.01) while no significant increase in both mortality outcomes was seen in treated HTN population. Similarly, after adjusting for variables in model 2, there was an increase in all-cause mortality (HR: 1.59, CI: 1.28–1.96, *p* < 0.01, Figure 2A) and CVD-related mortality (HR: 2.36, CI: 1.36–4.10, *p* < 0.01, Figure 2B) in untreated HTN. In treated HTN however, a lower magnitude of the effect was seen in all-cause mortality (HR: 1.26, CI: 1.03–1.55, *p* = 0.03, Figure 2A) and a non-significant increase was demonstrated in CVD-related mortality (HR: 1.51, CI: 0.87–2.63, *p* = 0.14, Figure 2B).

## Discussion

Both HTN and NAFLD share common risk factors and are both a major public health concern but there remains a low awareness of the bidirectional links between the two diseases (18, 21). Previous studies have shown NAFLD to be associated with an increased risk of HTN (35) and up to 40% of patients with NAFLD worldwide are estimated to be hypertensive (2). However, evidence on the impact of HTN in NAFLD remains scarce. The present study thus analyzed population-level data of 11,435 individuals with NAFLD. which found that 45.65% were affected by HTN. Individuals with NAFLD who are of older age, diabetic, dyslipidemic, and female were more likely to be associated with HTN. The presence of HTN increased the risk of all-cause and cardiovascular mortality in NAFLD but a sensitivity analysis showed that NAFLD patients with uncontrolled HTN had a significantly increased risk of CVD and all-cause mortality while controlled HTN had a lower magnitude of increase in all-cause mortality and non-significant increase in CVD mortality.

The act of screening and control of HTN is simple in clinical practice and can potentially attenuate the course of NAFLD. The inflammatory milieu in NAFLD potentiates hepatocyte injury and releases damage-associated molecular patterns in the circulation thereby triggering chronic inflammation. Inflammation is closely associated with the sympathetic system which modulates the activation of the renin-angiotensin system (RAS) in the maintenance of blood pressure. Similarly, the RNA-sequence and microarray dataset from gene cards also show a higher expression of the RAS components in patients with NAFLD. Additionally, insulin resistance, oxidative stress, and circulation of advanced glycation end products (AGE) contribute to vascular aging resulting in a sustained increase in blood pressure (20).

While HTN is conventionally thought to be more prevalent in men, our study shows that women with NAFLD were more likely to have HTN. The results could be explained by the presence of older women who are more likely to be postmenopausal resulting in worsened vascular function and increased susceptibility for HTN (36). However, the female gender in NAFLD is also related to a higher risk of advanced fibrosis (37) which may result in increased arterial stiffness and endothelial dysfunction (38). The assessment for NAFLD at the population level generally requires steatosis for diagnosis and fibrosis markers for severity assessment (39, 40). Due to the data availability in NHANES, fibrosis was assessed by FIB-4 which has been validated to be the most accurate blood-based fibrosis assessment for population studies (30).

In NAFLD, current guidelines by the AASLD (26) emphasized the importance of HTN as part of the initial assessment for comorbidities. However, there appears to be a lack of specific recommendations for the management of HTN in NAFLD (26). It is, however, important to note that despite the presence of a bidirectional relationship, current guidelines by the American Heart Association (AHA) (17) and European Society of Hypertension (ESH) (31) have yet to recognize NAFLD as a potential cause of HTN and the awareness of NAFLD remains low amongst cardiologists (41). While lifestyle modifications serve as a forefront in the care of NAFLD (8, 42) and HTN, these interventions are often unsuitable (43, 44). Instead, ACE inhibitors may be considered for use in individuals with NAFLD due to the multifactorial benefits they present where aside from regulation of blood pressure (45), ACE inhibitors have been found to reduce decompensation (46), hepatic fibrosis (47), and steatosis (48) in NAFLD and may also provide protection against CKD (49).

## Limitations

The main strength of this study is the assessment of longitudinal outcomes in a large cohort of multi-ethnic patients from NHANES that allowed for a comprehensive assessment of clinical burden in NAFLD. However, there are several limitations noted in our study. The population blood pressure was measured during a single timepoint and may not accurately capture the variability in blood pressure. Additionally, the self-reported alcohol usage during healthcare visits was subjected to recall bias but is however a known limitation in the definition of NAFLD. Lastly, we are unable to ascertain the definitive indication for antihypertensives.

## Conclusion

Hypertension (HTN) remains a major comorbidity in NAFLD, and the current study brings forth evidence for the need for better blood pressure regulation amidst the growing burden of NAFLD globally. Importantly, HTN can increase the risk of overall mortality and cardiovascular mortality if left unchecked although prompt treatment can reduce the risk of events. There remains a need for institutionalized screening of HTN in NAFLD and awareness building for multidisciplinary care.

## Data availability statement

Publicly available datasets were analyzed in this study. This data can be found here: https://wwwn.cdc.gov/nchs/nhanes/.

## Ethics statement

Ethical review and approval was not required for the study on human participants in accordance with the local legislation and institutional requirements. Written informed consent for participation was not required for this study in accordance with the national legislation and the institutional requirements.

## Author contributions

Conceptualization and design: CN, NC, and MM. Acquisition of data and analysis and interpretation of data: CN, ZW, NC, KC, JX, NS, WL, DT, RL, PT, JY, and GK. Writing the original draft: CN, ZW, NC, and KC. Writing, reviewing, and editing: DH, J-WW, MC, MD, NT, MN, MS, AS, and MM. All authors approve the final version of the manuscript, including the authorship list and agree to be accountable for all aspects of the work in ensuring that questions related to the accuracy or integrity of any part of the work are appropriately investigated and resolved.

## Conflict of interest

Author AS is President of Sanyal Biotechnology and has stock options in Genfit, Akarna, Tiziana, Indalo, Durect, and Galmed. He has served as a consultant to Astra Zeneca, Nitto Denko, Enyo, Ardelyx, Conatus, Nimbus, Amarin, Salix, Tobira, Takeda, Jannsen, Gilead, Terns, Birdrock, Merck, Valeant, Boehringer-Ingelheim, Lilly, Hemoshear, Zafgen, Novartis, Novo Nordisk, Pfizer, Exhalenz, and Genfit. He has been an unpaid consultant to Intercept, Echosens, Immuron, Galectin, Fractyl, Syntlogic, Affimune, Chemomab, Zydus, Nordic Bioscience, Albireo, Prosciento, Surrozen, and Bristol Myers Squibb. His institution has received grant support from Gilead, Salix, Tobira, Bristol Myers, Shire, Intercept, Merck, Astra Zeneca, Malinckrodt, Cumberland, and Norvatis. He receives royalties from Elsevier and UptoDate. Author MN has been on the advisory board for 89BIO, Gilead, Intercept, Pfizer, Novo Nordisk, Blade, EchoSens, Fractyl, Terns, Siemens, and Roche diagnostic; He has received research support from Allergan, BMS, Gilead, Galmed, Galectin, Genfit, Conatus, Enanta, Madrigal, Novartis, Pfizer, Shire, Viking, and Zydus. He is a minor shareholder or has stocks in Anaetos, Rivus Pharma, and Viking. Author MC has received speaker's fees and research grants Astra Zeneca, Abbott Technologies, and Boston Scientific. The remaining authors declare that the research was conducted in the absence of any commercial or financial relationships that could be construed as a potential conflict of interest.

## Publisher's note

All claims expressed in this article are solely those of the authors and do not necessarily represent those of their affiliated organizations, or those of the publisher, the editors and the reviewers. Any product that may be evaluated in this article, or claim that may be made by its manufacturer, is not guaranteed or endorsed by the publisher.
